# A 3-Ligament Syndesmotic Injury Is at Higher Risk for Malreduction Than a 2-Ligament Injury: A CT-Based Analysis

**DOI:** 10.1177/10711007241238227

**Published:** 2024-05-30

**Authors:** Fabian Tobias Spindler, Wolfgang Böcker, Hans Polzer, Sebastian Felix Baumbach

**Affiliations:** 1 Department of Orthopaedics and Trauma Surgery, Musculoskeletal University Center Munich (MUM), University Hospital, LMU Munich, Munich, Germany

**Keywords:** syndesmosis, malreduction, suture-button system, CT, quality of reduction, syndesmotic injury

## Abstract

**Background:**

Syndesmotic malreduction is common and has been associated to an impaired outcome. Various risk factors for DTFJ malreduction have been postulated. The aims of this study were to assess the DTFJ malreduction rate based on (1) the severity of the syndesmotic injury, (2) the anatomy of the tibial incisura, and (3) the fixation device used in patients treated with suture-button systems.

**Methods:**

This retrospective, radiographic study included all adult patients who were treated for an acute, unilateral, and unstable syndesmotic injury with a suture-button system and postoperative bilateral CT imaging. Included were isolated syndesmotic injuries and fracture cases. The number of syndesmotic ligaments injured, that is, 2-ligament (AiTFL+IOL) and 3-ligament (AiTFL+IOL+PiTFL), was rated for each patient. The quality of DTFJ reduction, as well as the anatomy of the tibial incisura, was rated based on the postoperative, bilateral CT images and the intraoperative DTFJ reduction was recalculated based on the drilling-tunnel deviation. The possible influence on the DTFJ malreduction rate was assessed.

**Results:**

A total of 147 patients were included, and 94 and 53 patients had a 2- and 3-ligament syndesmotic injury, respectively. In addition, 113 patients were treated with a single-button system, 26 with a double suture-button system, and 8 with a hybrid fixation (suture-button + screw). Malreduction was significantly higher in 3-ligament compared with 2-ligament injuries, both intraoperatively (51% vs 27%; *P* = .003) and postoperatively (28% vs 11%; *P* = .006). The tibial anatomy had no significant influence on the malreduction rates. No significant differences were seen per the different fixation devices used independent of the number of ligaments injured.

**Conclusion:**

This study did not find an influence of the incisura’s anatomy on the DTFJ malreduction rate. However, we did find that 3-ligament syndesmotic injuries carried a higher risk of intra- and postoperative malreduction compared with 2-ligament injuries.

## Graphical abstract

**Graphical Abstract:**
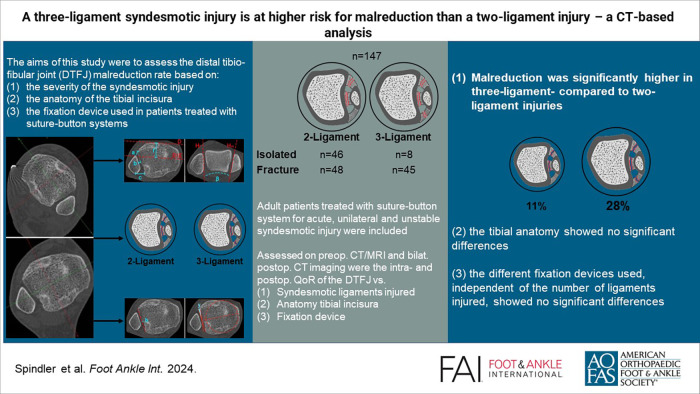
This is a visual representation of the abstract.

**Level of Evidence:** Level III, retrospective radiologic study.

## Introduction

The syndesmotic complex resembles a dynamic 3-point fixation of the fibula to the tibia. The 3 distinct portions of the syndesmosis are the anterior inferior tibiofibular ligament (AiTFL), the interosseous ligament (IOL), and the posterior inferior tibiofibular ligament (PiTFL).^37^ Syndesmotic injuries can occur isolated, in course of an ankle sprain, or in combination with an ankle fracture.

Various classification systems have been published for syndesmotic injuries.^29^ They are usually classified into stable, latent unstable, and frank diastasis.^32^ The authors favor a classification per the number of syndesmotic ligaments ruptured.^16^ An isolated injury to the AiTFL is considered a stable injury; a rupture of the AiTFL and IOL results in a subtle translational and rotational instability of the fibula and thus a latent instability of the distal tibiofibular joint (DTFJ). An additional injury to the PiTFL results in a complete instability of the DTFJ, which may be identified on plain radiographs as a frank diastasis.^2^

There is an ongoing discussion on how to treat unstable syndesmotic injuries. Irrespective of the fixation device used, the predominant goal is the anatomical reduction of the DTFJ. A malreduction of the DTFJ is known to result in pain and osteoarthritis.^1,15,26^ Consequently, it is of great importance to identify possible risk factors for malreduction. Various factors have been postulated to be associated to DTFJ malreduction. Three highly discussed risk factors are the degree of syndesmotic instability, the anatomic variance of the tibial incisura, and the fixation device used. It appears reasonable that a 3-ligament (AiTFL+IOL+PiTFL) injury is more likely to be malreduced than a 2-ligament injury (AiTFL+IOL) as a result of the increased DTFJ instability.^23^ Moreover, previous studies were able to show a high variance of the tibial incisura, especially its depth, engagement, and rotation and a possible association to DTFJ malreduction.^4,5^ Finally, the devices for DTFJ, that is, a single suture-button, double suture-button, or suture-button and syndesmotic screw, could also influence the quality of DTFJ reduction. The authors are not aware of any study concisely assessing the influence of these factors on DTFJ malreduction, especially if the DTFJ was stabilized by a suture-button system.

The aims of this study were to assess the DTFJ malreduction rate based on (1) the severity of the syndesmotic injury, (2) the anatomy of the tibial incisura, and (3) the fixation device used in patients treated with suture-button systems.

## Materials and Methods

This retrospective, radiographic study was conducted on a previously published cohort^30^ and was a priori approved by the local ethics committee (no. 21-1136).

### Patient Selection

The patient selection process as well as the surgical procedures at the authors’ institution has been described in detail before.^30^ In brief, 147 adult patients (≥18 years) who had been treated with a suture-button system for an acute, unilateral ankle injury met the study criteria. The type of injury (ie, isolated syndesmotic injury or ankle fracture) was of no matter. Patients must have received a postoperative, bilateral CT. Patients with preexisting injuries to the contralateral ankle or concomitant injuries outside of the ankle joint were excluded from further analysis.

The surgical procedure was the same for all patients. In case of a concomitant fracture, the syndesmotic complex was addressed following open reduction and internal fixation of all bony injuries. A fracture to the posterior malleolus was also treated by open reduction and internal fixation if of sufficient size to fix it with at least 1 screw. Fibular length and axial stability of the fractures was restored in all cases. In all cases, the DTFJ was reduced using a ball-clamp in a center-center alignment with the ankle in 90-degree dorsiflexion.^6^ Center-center refers to the center of the medial and lateral malleolus at the height of the ankle joint.^17^ Stabilization was performed either by a single suture-button system, double suture-button system, or a single suture-button system and a syndesmotic screw. The suture-button system used in all cases was the TightRope (Arthrex, Naples, FL). Syndesmotic fixation was performed per the treating surgeon’s preference.

### Data Assessment

General data gathered included demographics, injury-specific details, and surgical information. The assessment of the primary points of interest, that is, the quality of DTFJ reduction intra- and postoperatively, the severity of the syndesmotic injury, the anatomy of the tibial incisura, and the fixation devices used, is outlined in the following.

#### Quality of DTFJ reduction

**Figure 1.**
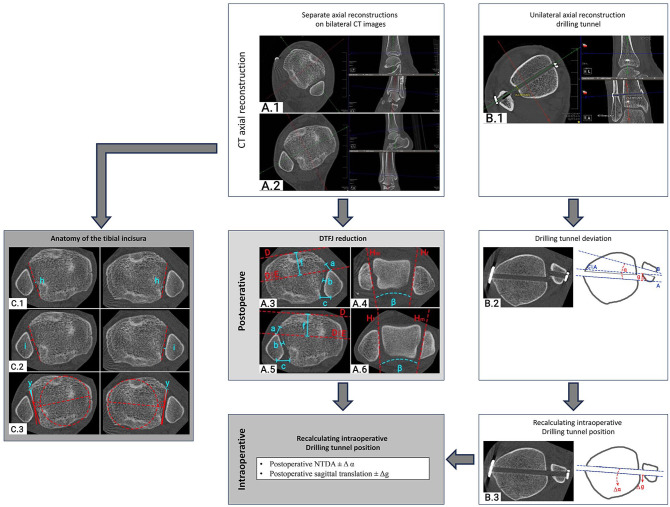
Quality assessment of the DTFJ reduction on bilateral computed tomography imaging. (A.1 and A.2) DTFJ reconstruction of both ankles; (A.3-A.6) measurements of the DTFJ reduction. (B.1 and B.2) Reconstruction of the drilling tunnel and its measurement; (B.3) illustration of the recalculated intraoperative DTFJ reduction. (C.1-C.3) Measurement of the anatomy of the tibial incisura. Labels: *a*, anterior tibiofibular distance; *b*, central tibiofibular distance; *c*, posterior tibiofibular distance; *D*, tangent to the anterior aspect of the tibia; *E*, translation of D to the most anterior part of the fibula; *f*, sagittal DTFJ translation; *H*, tangent to the medial malleolus (*H_m_*) or fibula (*H_f_*); *β*, NTDA; *A*, tangent of the posterior tibial drilling tunnel border; *C*, tangent of the anterior tibial drilling tunnel border; *B*, tangent of the anterior fibular drilling tunnel border; *α*, angulation/rotation of the drilling tunnel; *g*, sagittal translation of the drilling tunnel; *h*, tibial incisura depth; *i*, fibula engagement; *y*, tibial incisura rotation. (DTFJ, distal tibiofibular joint; NTDA, Nault talar dome angle.)

The post- and intraoperative quality of reduction (QoR) was assessed on postoperative, bilateral CT images by 2 independent authors (F.T.S., S.F.B.), as described in detail previously,^30^ and is outlined in Figure 1A and 1B. The postoperative QoR of the DTFJ was assessed on separately reconstructed axial CT slices, bilaterally (Figure 1A.1 and 1A.2). The measurements conducted (Figure 1A.3-1A.6) and the physiological cutoff values were the modified sagittal translation (≤2 mm); the Nault talar dome angle (NTDA; ≤10°); and the anterior (≤2 mm), central (≤1.5 mm), and posterior (≤2 mm) tibiofibular distances.^18,25^ Postoperative malreduction was defined as any parameter deviating between the injured and uninjured side for more than the physiological cutoff values.^18,25^ The values analyzed are the result of the observed side difference.

Next, the drilling tunnel of the suture-button system was reconstructed at its full length (Figure 1B.1). When 2 devices were used, only the distal drilling tunnel was reconstructed, as this drilling tunnel was always placed first. Based on the axial reconstruction, the rotational and translational deviation of the fibular compared with the tibial drilling tunnels were measured (Figure 1B.2). As the fibular and tibial drilling tunnels must have been aligned intraoperatively, the intraoperative reduction of the injured side was recalculated by adding or subtracting the drilling tunnel deviation from the postoperative measurements (modified sagittal translation and the Nault talar dome angle; Figure 1B.3). Based on these adjusted measurements, the intraoperative malreduction was calculated, again using the above outlined cutoff criteria and providing the product of the difference.

##### Severity of the syndesmotic injury

**Figure 2.**
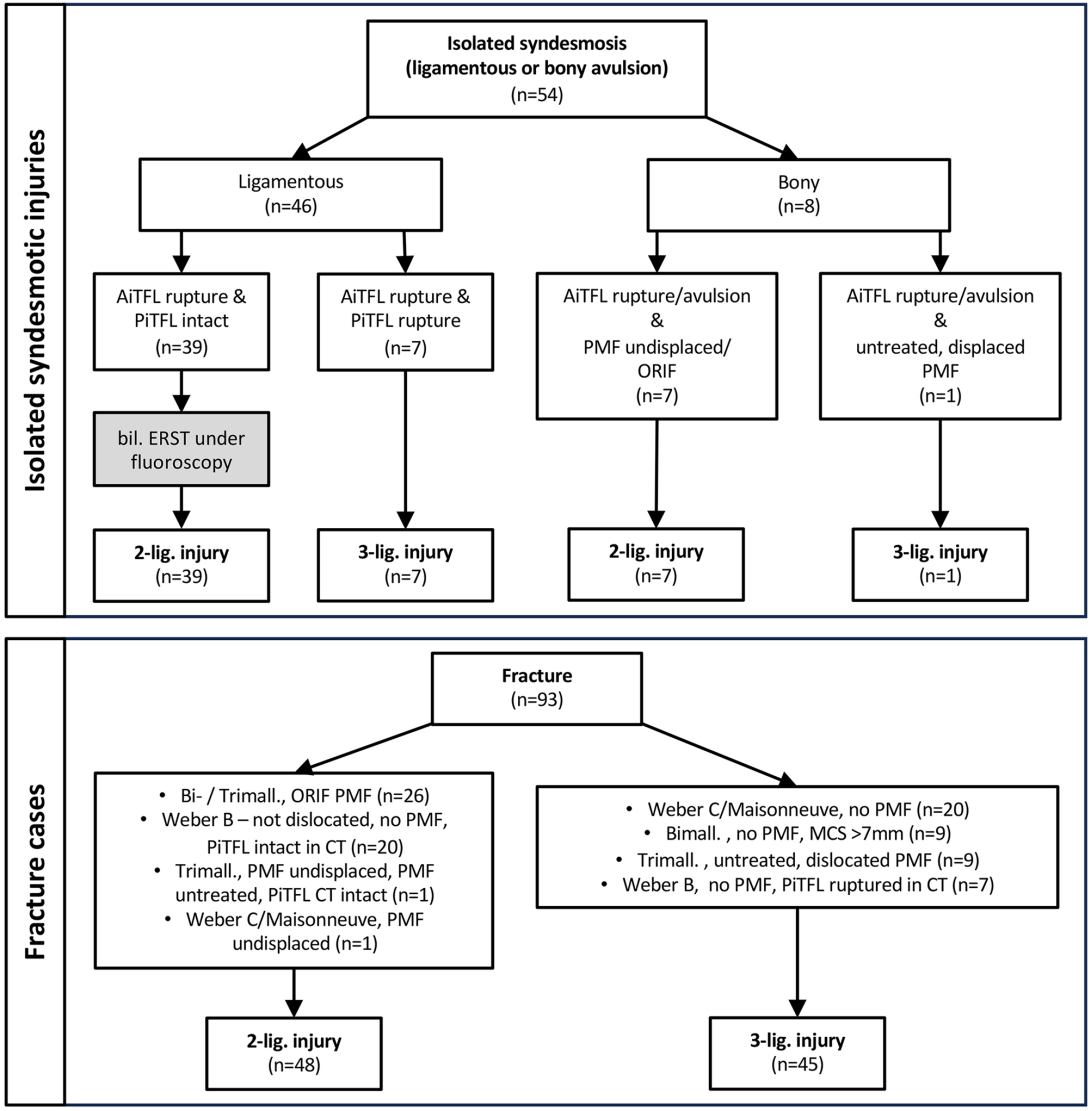
Classification of the injuries to the syndesmotic complex. AiTFL, anterior-inferior tibiofibular ligament; bil., bilateral; Bimall., bimalleolar fracture; CT, computed tomography; ERST, external rotation stress test; lig., ligament; MCS, medial clear space; n, number; ORIF, open reduction and internal fixation; PiTFL, posterior-inferior tibiofibular ligament; PMF, posterior malleolar fracture; Trimall., trimalleolar fracture.

Each injury was classified per the number of syndesmotic ligaments injured into 2-ligament (AiTFL+IOL) and 3-ligament (AiTFL+IOL+PiTFL) injuries (Figure 2). Isolated syndesmotic injuries were rated based on magnetic resonance imaging. The AiTFL and PiTFL were assessed, and if both were ruptured, it was classified as a case of 3-ligament injury. In case the PiTFL was intact but the AiTFL ruptured, a bilateral external rotation stress test under fluoroscopy was performed. Any increased medial clear space widening compared to the contralateral, uninjured side was rated as a 2-ligament injury. In case of no widening, the injury was classified as an isolated AiTFL injury and treated conservatively.^29^ In fracture cases, the classification was based on the situation present at the time of stabilization of the DTFJ, that is, after fixing all bony injuries. The classification was based on the preoperative CT imaging and treatment details. In doubtful cases, the PiTFL was rated on the preoperative CT images.^31^ A Weber C fracture without a fracture to the posterior malleolus was rated as a 3-ligament injury. If an open reduction and internal fixation of a posterior malleolus fracture was performed, the case was classified as a 2-ligament injury.

##### Anatomy of the tibial incisura

The anatomy of the tibial incisura was again analyzed on the postoperative bilateral axial CT slices. The reconstruction process and measurement location were the same as for the QoR assessment. Two independent authors (F.T.S., S.F.B.) conducted the following measurements: incisura depth (Figure 1C.1), fibula engagement (Figure 1C.2), and incisura rotation (Figure 1C.3). These measurements were conducted per the recommendations of Boszczyk et al.^4,5^

##### Fixation device used

The patient records and postoperative images were screened for the fixation device used. Per the inclusion criteria, patients were treated with either of the following setups: single suture-button, double suture-button, or suture-button and syndesmotic screw.

### Data Analysis and Statistics

Per the aim of the study, the influence of (1) the severity of the syndesmotic injury, (2) the anatomy of the tibial incisura, and (3) the fixation setup used on the DTFJ malreduction rate (percentage DTFJ malreduction, each parameter separately) was assessed.

Data were analyzed using the jamovi project (jamovi, version 2.3, 2023). Descriptive statistics, independent samples *t* test, χ^2^ test, as well as an analysis of variance were conducted. In case of statistically significant differences regarding the analysis of variance, a post hoc test was conducted. If not stated differently, values are given as mean ± SD. *P* values lower than .05 were considered statistically significant.

## Results

The mean age of the patient population was 39 ± 15 years, 34% were female, and the mean body mass index was 26.3 ± 4.8. The left side was injured in 50% of the patients. The primary injury was an ankle fracture in 63%. A total of 94 patients (64%) suffered a 2-ligament injury and 53 patients (36%) a 3-ligament injury (Figure 2). Overall, 113 patients were treated with a single suture-button, 26 with a double suture-button, and 8 with a suture-button and syndesmotic screw. Neither the patient’s age (*P* = .838), sex (*P* = .816), or body mass index (*P* = .757) differed significantly between anatomically or malreduced DTFJ cases.

### Influence of the Number of Syndesmotic Ligaments Injured

Overall, 3-ligament injuries resulted in a significantly higher postoperative DTFJ malreduction rate compared with 2-ligament injuries (28% vs 11%; *P* = .006; Table 1). Sagittal malreduction, that is, anterior-to-posterior fibular translation, was the most common direction of malreduction, both postoperatively and intraoperatively. Malreduction rates in 2- and 3-ligament injuries significantly decreased from intraoperatively (51% and 27%) to postoperatively (28% and 11%).

**Table 1.** Analysis of Malreduction Rate per the Number of Syndesmotic Ligaments Injured.^a^

	Postoperative Quality of Reduction						Intraoperative Quality of Reduction		
	Malreduction, n (%)	Δ Anterior Distance^b^, mm	Δ Central Distance^b^, mm	Δ Posterior Distance^b^, mm	Δ NTDA^b^, degrees	Δ AP Translation^b^, mm	Malreduction n (%)	Δ DTFJ Rotational Reduction, degrees	Δ AP Translational Reduction, mm
2-ligament injury (n = 94)	**10 (11)**	1.0 ± 0.7	0.8 ± 0.6	**1.0** **±** **0.8**	3.8 ± 3.4	**1.1** **±** **0.7**	**25 (27)**	4.6 ± 4.4	**1.3** **±** **0.8**
3-ligament injury (n = 53)	**15 (28)**	1.3 ± 1.0	0.6 ± 0.4	**1.4** **±** **1.1**	3.4 ± 3.6	**1.7** **±** **1.4**	**27 (51)**	4.4 ± 4.6	**2.1** **±** **1.7**
*P* value	**.006**	.074	.168	**.031**	.428	**<.001**	**.003**	.780	**<.001**

Abbreviations: AP, anterior-posterior; intraop., intraoperative; NTDA, Nault talar dome angle; postop., postoperative.

a Boldface indicates significant difference (*P* < .05).

b Values given as a product of the difference between the native and operated side.

### Influence of the Anatomy of the Tibial Incisura

The injured side mean incisura depth was 3.8 ± 1.3 mm, mean fibula engagement 0.7 ± 1.4 mm with 32 patients (22%) being disengaged, and mean incisura rotation was 5.8 ± 4.6 degrees, with 12 patients (8%) being anteriorly opened. The anatomy of the tibial incisura showed significant correlation between the injured and uninjured side (incisura depth: Pearson *r* = 0.771, *P* < .001; fibula engagement: Pearson *r* = 0.519, *P* < .001; incisura angulation: Pearson *r* = 0.609, *P* < .001).

Overall, no significant differences between the tibial anatomy and the intra- and postoperative QoRs were observed (Table 2). This stayed true for overall analysis as well as ligament-specific analysis.

**Table 2.** Analysis of the Influence of the Anatomy of the Tibial Incisura on the Malreduction Rate, Overall and Separately for 2- and 3-Ligament Injuries.

	Postoperative Quality of Reduction				Intraoperative Quality of Reduction			
Ligaments Injured	Reduction, n (%)	Incisura Depth, Mean±SD, mm	Fibula Engagement, Mean±SD, mm	Incisura Rotation, Mean±SD, degrees	Reduction, n (%)	Incisura Depth, Mean±SD, mm	Fibula Engagement, Mean±SD, mm	Incisura Rotation, Mean±SD, degrees
Overall (n = 147)								
Anatomical	122 (83)	3.8 ± 1.4	0.6 ± 1.4	5.7 ± 4.4	95 (65)	3.8 ± 1.4	0.6 ± 1.4	5.8 ± 4.4
Malreduced	25 (17)	3.7 ± 1.1	0.9 ± 1.3	6.7 ± 5.4	52 (35)	3.7 ± 1.2	0.8 ± 1.2	5.9 ± 4.8
* P* value		.841	.383	.300		.870	.388	.952
2-ligament injury (n = 94)								
Anatomical	84 (89)	3.8 ± 1.5	0.7 ± 1.3	5.9 ± 4.5	69 (73)	3.8 ± 1.5	0.7 ± 1.3	5.8 ± 4.6
Malreduced	10 (11)	4.2 ± 0.7	1.4 ± 1.3	7.5 ± 4.6	25 (27)	3.9 ± 1.1	1.0 ± 1.2	6.7 ± 5.4
* P* value		.472	.142	.337		.831	.358	.467
3-ligament injury (n = 53)								
Anatomical	38 (72)	3.7 ± 1.0	0.3 ± 1.6	5.2 ± 4.2	26 (49)	3.6 ± 0.9	0.2 ± 1.7	5.8 ± 4.0
Malreduced	15 (28)	3.4 ± 1.2	0.6 ± 1.3	6.2 ± 4.0	27 (51)	3.6 ± 1.3	0.6 ± 1.2	5.2 ± 4.2
* P* value		.446	.614	.391		.868	.340	.586
Overall *P* value		.315	.490	.798		.798	.830	.382

Abbreviations: n, number; mm, millimeter; °, degree of angulation.

### Influence of the syndesmotic fixation device

Overall, no significant differences between the different fixation techniques were found for any of the assessed malreduction parameters (Table 3). Due to the significant differences in malreduction rates in 2- and 3-ligament injuries, the possible influence of the fixation technique on the malreduction rate was recalculated separately for a single- and double suture-button system. The number of patients treated with a suture-button system and a syndesmotic screw per group was too small for a statistical comparison. Again, no relevant differences could be found.

**Table 3.** Analysis of the Influence of the Syndesmotic Fixation Device on the Malreduction Rate, Overall and Separately for 2- and 3-Ligament Injuries.^a^

		Postoperative Quality of Reduction						Intraoperative Quality of Reduction		
Ligaments Injured	Syndesmotic Treatment	Malreduction, n (%)	Δ Anterior Distance^b^, mm	Δ Central Distance^b^, mm	Δ Posterior Distance^b^, mm	Δ NTDA^b^, degrees	Δ AP Translation^b^, mm	Malreduction, n (%)	Δ DTFJ Rotational Reduction, degrees	Δ AP Translational Reduction, mm
Overall (n = 147)	Single suture-button (n = 113)	20 (18)	1.1 ± 0.9	0.7 ± 0.5	1.1 ± 1.0	3.6 ± 3.4	1.4 ± 1.1	41 (36)	4.4 ± 4.4	1.6 ± 1.3
	Double suture-button (n = 26)	3 (12)	1.0 ± 0.8	0.8 ± 0.6	1.2 ± 1.2	3.2 ± 2.9	1.0 ± 0.6	9 (35)	4.8 ± 4.3	1.5 ± 1.0
	Suture-button + Syndesmotic screw (n = 8)	2 (25)	0.9 ± 0.8	0.6 ± 0.4	1.7 ± 2.2	5.6 ± 5.4	1.6 ± 1.6	2 (25)	5.3 ± 6.3	1.1 ± 1.0
	*P* value	.621	.493	.748	.991	.458	.532	.809	.865	.378
2-ligament (n = 92)	Single suture-button (n = 76)	8 (11)	1.0 ± 0.8	0.7 ± 0.5	1.0 ± 1.0	4.0 ± 3.6	1.1 ± 0.7	19 (25)	4.6 ± 4.6	1.32 ± 0.9
	Double suture-button (n = 16)	2 (13)	0.9 ± 0.6	1.1 ± 0.7	1.2 ± 1.3	3.4 ± 2.6	0.8 ± 0.6	6 (38)	4.6 ± 3.5	1.26 ± 0.8
	*P* value	.818	.780	.935	.201	.586	.103	.307	.959	.796
3-ligament (n = 47)	Single suture-button (n = 37)	12 (32)	1.3 ± 1.1	0.7 ± 0.5	1.4 ± 0.8	3.0 ± 3.0	1.8 ± 1.5	22 (59)	3.9 ± 3.9	2.29 ± 1.8
	Double suture-button (n = 10)	1 (10)	1.2 ± 1.0	0.5 ± 0.4	1.1 ± 0.9	2.8 ± 3.5	1.4 ± 0.6	3 (30)	5.1 ± 5.6	1.84 ± 1.1
	*P* value	.159	.827	.872	.534	.827	.364	.098	.419	.460
*P* value		n.a.	.924	**.048**	.206	.852	.761	.873	.472	.509
2-ligament (n = 2)	Suture-button + Syndesmotic screw (n = 2)	0 (0)	0.7 ± 0.4	0.3 ± 0.1	0.7 ± 0.8	2.9 ± 1.4	1.1 ± 1.3	0 (0)	2.8 ± 1.6	0.95 ± 1.1
3-ligament (n = 6)	Suture-button + Syndesmotic screw (n = 6)	2 (30)	1.0 ± 0.9	0.7 ± 0.4	2.0 ± 2.52	6.5 ± 8.1	1.8 ± 1.8	2 (30)	6.1 ± 7.2	1.18 ± 1.1

Abbreviations: AP, anterior-posterior; intraop., intraoperative; NTDA, Nault talar dome angle; postop., postoperative.

a Boldface indicates significant difference (*P* < .05).

b Values given as a product of the difference between the native and operated side.

## Discussion

The aims of this study were to assess the DTFJ malreduction rate based on (1) the severity of the syndesmotic injury, (2) the anatomy of the tibial incisura, and (3) the fixation device used in patients treated with suture-button systems.^30^ DTFJ malreduction has received increasing attention, as it has been associated to significantly lower PROMs and higher rates of osteoarthritis.^33^ Therefore, identifying risk factors for malreduction is of importance. Only this knowledge will allow to decrease the rate of malreduction rates and therefore improve the outcome of our patients.

The overall malreduction rate of 17% is on the high side compared with previous studies, which have reported malreduction rates for suture-button systems ranging from 1% to 15%.^27,28^ Previous clinical studies have reported worse clinical outcomes for 3- compared to 2-ligament syndesmotic injuries.^9^ The present study assessed 3 commonly discussed factors possibly affecting DTFJ malreduction.

The first factor analyzed was the degree of DTFJ instability, based on the number of syndesmotic ligaments injured, that is, 2-ligament vs 3-ligament injuries. The authors are not aware of any clinical study analyzing this association. The degree of syndesmotic instability was the only factor associated to the malreduction rated. A significantly higher post- and intraoperative malreduction rate was found for 3-ligament (AiTFL, IOL, PiTFL) compared to 2-ligament syndesmotic injuries (AiTFL, IOL): 28% vs 11%, *P* = .006. Biomechanical studies were able to show an increase in DTFJ instability between 2- and 3-ligament dissection cases for coronal,^11-13,36^ sagittal,^19^ and rotational^36^ instability. This higher degree of instability is likely the reason for the increased malreduction rate in 3-ligament injuries observed herein. Technical factors that can help to avoid DTFJ malreduction are the padding of the shank,^3^ the placement of and the force applied to reduction clamp,^21,23^ as well as open reduction of the DTFJ.^22,24,38^

The second factor analyzed was the influence of the anatomy of the incisura and the engagement of the fibula. Previous studies were able to show an astonishing intersubject variability for the anatomy of the tibial incisura.^5,7,10^ It appears reasonable that a flat anteriorly angulated incisura could more easily result in an anteriorly malreduced DTFJ compared to a deep-socket incisura. Still, only a very few studies have so far assessed the influence of this anatomical variance on the malreduction rate.^5,7^ Cherney et al^7^ found a significant influence of the depth of the incisura on DTFJ malreduction in 35 patients. Boszczyk et al,^5^ whose methodology was applied herein, analyzed 72 ankle fracture patients with an unstable syndesmotic injury. They reported an association between a deep incisura and unengaged fibula for overcompression, as well as anteverted and retroverted incisurae for anterior and posterior fibula translation, respectively. Contrary to these studies, the present analysis revealed no influence of the incisura’s anatomy on the DTFJ malreduction rate. One reason could be the “natural correction potential” of the suture buttons used, as outlined in the following. It can be hypothesized, that this compensational mechanism outweighs, and therefore disguises, the effect of the anatomy of the incisura on DTFJ malreduction. Still, also for the intraoperative values, no significant differences per the anatomy of the incisura on the malreduction rate could be found.

The final factor analyzed was the fixation devices used. The fixation constructs used were a single suture-button system, 2 suture-button systems, or the combination of a suture-button system and a syndesmotic screw. Previous biomechanical studies showed a varying sagittal and coronal plane stability for different syndesmotic stabilization devices. Screws are more stable than a double suture-button or a single suture-button construct.^8,20,35,39^ The authors had therefore hypothesized, that the syndesmotic fixation technique might have an influence on the malreduction rate. Still, neither the number of suture-button devices nor the combination of suture-button systems and a syndesmotic screw had an influence on the DTFJ malreduction rate. This was independent of the type of syndesmotic lesion (ie, 2- vs 3-ligament injuries) and the intra- and postoperative DTFJ malreduction rate.

However, the malreduction rate significantly varied between intra- and postoperatively. Previous studies have reported on the so-called “natural correction potential” of suture-button systems.^14,30,34^ Westermann et al^34^ conducted biomechanical studies in which the DTFJ was malreduced intentionally. It was then stabilized using a suture-button system, and the natural correction potential was assessed. They reported a sagittal correction potential of 1 mm for anteriorly and 6.7 mm for posteriorly malreduced DTFJs. We are only aware of one other clinical study analyzing the “natural correction potential,” which reported sagittal drilling tunnel deviations of 1.2 ± 1.4 mm with a maximum correction of 5.7 mm.^14^ These reported values are well in line with our observed sagittal drilling tunnel deviation of 0.9 ± 0.8 mm with a maximum of 4.3 mm.^30^ Previous studies were able to show that this “natural correction potential” compensates predominantly toward a more anatomical reduced DTFJ.^14,30,34^ The flexible nature of the suture-button system, along with the persisting tension of the intact soft tissue structures and mortise congruency due to anatomical osseous reduction, therefore apparently compensates for an intraoperative DTFJ malreduction. This most likely explains the reported lower DTFJ malreduction rates of suture-button systems (1% to 15%)^27,28^ compared with that of syndesmotic screws (11.5% and 39%). On the other hand, the “natural correction potential” of suture-button systems can only compensate malreduction to a certain extent. This might explain why the degree of compensation did not differ between the more stable 2-ligament and the more unstable 3-ligament syndesmotic injuries in the current study.

The study had several limitations, which must be discussed. First of all, the current study assessed the influence of the patients’ age, sex, body mass index, type of injury (isolated syndesmosis vs fracture), anatomy of the tibial incisura, fixation device, and number of syndesmotic ligaments injured. Despite the considerable number of parameters assessed, further confounders could possibly influence the malreduction rate. These include the type of fracture, quality of fracture reduction, and type of DTFJ reduction. DTFJ reduction can be performed open, that is, by direct visualization, or closed. Moreover, the reduction itself can be performed manually or with the help of a reduction clamp. The accuracy of the positioning of the reduction clamp and the pressure applied again possibly influence the DTFJ reduction quality. Although some of these parameters were controlled for, such as clamp positioning, future studies are needed to assess the effect of further factors possibly influencing the quality of DTFJ reduction. Second, this study was based on a retrospective data set. Still, because only radiographic parameters were analyzed, the retrospective study design most likely did not result in a relevant selection bias. Finally, the classification per the number of syndesmotic ligaments injured is a source of error. Whereas the ligamentous syndesmotic injuries could clearly be classified by magnetic resonance imaging, some fracture cases were not that easy to be grouped. In those cases, the PiTFL was rated on the CT images, which has been shown to be feasible.^31^

Despite these limitations, the study has several strengths. It is the first study to systematically assess the potential influence of different stages of syndesmotic instability on the postoperative quality of reduction in patients treated with a suture-button system. Moreover, it is a single center study and a uniform reduction technique for the DTFJ was applied. Finally, the number of patients did allow for a thorough analysis of different subgroups.

In conclusion, we did not find an influence of the incisura’s anatomy on the DTFJ malreduction rate. Notably, 3-ligament syndesmotic injuries bear a higher risk of intra- and postoperative malreduction compared to 2-ligament injuries. In cases of a complete syndesmotic disruption, surgeons should take precautionary measures to ensure an anatomical reduction of the distal tibiofibular joint.

## Supplemental Material

sj-pdf-1-fai-10.1177_10711007241238227 – Supplemental material for A 3-Ligament Syndesmotic Injury Is at Higher Risk for Malreduction Than a 2-Ligament Injury: A CT-Based Analysis Supplemental material, sj-pdf-1-fai-10.1177_10711007241238227 for A 3-Ligament Syndesmotic Injury Is at Higher Risk for Malreduction Than a 2-Ligament Injury: A CT-Based Analysis by Fabian Tobias Spindler, Wolfgang Böcker, Hans Polzer and Sebastian Felix Baumbach in Foot & Ankle International
